# Everyday bat vocalizations contain information about emitter, addressee, context, and behavior

**DOI:** 10.1038/srep39419

**Published:** 2016-12-22

**Authors:** Yosef Prat, Mor Taub, Yossi Yovel

**Affiliations:** 1 Department of Zoology, Faculty of Life sciences, Tel Aviv University, Tel Aviv, Israel

## Abstract

Animal vocal communication is often diverse and structured. Yet, the information concealed in animal vocalizations remains elusive. Several studies have shown that animal calls convey information about their emitter and the context. Often, these studies focus on specific types of calls, as it is rarely possible to probe an entire vocal repertoire at once. In this study, we continuously monitored Egyptian fruit bats for months, recording audio and video around-the-clock. We analyzed almost 15,000 vocalizations, which accompanied the everyday interactions of the bats, and were all directed toward specific individuals, rather than broadcast. We found that bat vocalizations carry ample information about the identity of the emitter, the context of the call, the behavioral response to the call, and even the call’s addressee. Our results underline the importance of studying the mundane, pairwise, directed, vocal interactions of animals.

Many animal species use vocal communication^1^, but the chaotic nature of the acoustics of these vocalizations often complicates their cataloging into clearly divided types and contexts^2^. Understanding the encapsulated information in animal vocalizations is central to the study of sociality, communication, and language evolution. Yet, in the research of nonhuman animals, the purpose and meaning of the vocal message often remain obscure. Researchers of animal communication, seeking homology to semantics, may relate behavioral observations to acoustic measurements, and thus reveal some of the information content of vocal expressions. Indeed, several studies have described cases of vocalizations as functionally referential, i.e. signals which are both specific to a certain context and elicit an appropriate response by a receiver^3^,^4^,^5^. However, the requisite for a specific response might be too strict, ignoring many other information bearing vocalizations. Many vocalizations evoke a response according to their context or their receiver’s internal state^6^, and not solely based on their acoustics. Thus, the information capacity of animal vocalizations should still be explored, even when interpreting the behavioral responses is not straightforward^7^. Identifying context specific calls can be a first step toward the recovering of meaning in animal communication. For example, many studies indicated the existence of context specific vocalizations in various taxa (e.g., birds^8^,^9^,^10^, cetaceans^11^,^12^, dogs^13^, bats^14^,^15^, and nonhuman primates^16^). Notably, the distinctive alarm calls of some species are acoustically unique to different types of danger^17^,^18^,^19^. Other studies have exemplified the ability to distinguish between emitting individuals based on acoustic features of their vocalizations^20^,^21^,^22^,^23^,^24^,^25^,^26^,^27^,^28^,^29^,^30^,^31^, while several studies, mainly in primates, revealed subtle context-dependent acoustic modifications of calls in everyday behaviors, such as food-related behaviors^32^, agonistic interactions^33^, and long-distance calls^29^.

Bats are social mammals that often roost in very large colonies, and commonly engage in social interactions. They rely heavily on vocalizations for social communication^14^,^27^,^34^, yet little is known about the function and informational content of these vocalizations. In this study, we used the extremely social and vocal Egyptian fruit bat (*Rousettus aegyptiacus*) to elucidate some of the huge potential of information capacity in a mammalian vocal communication system. Egyptian fruit bats are social mammals, that aggregate in groups of dozens to thousands of individuals, can live to the age of at least 25 years^35^, and are capable of vocal learning^36^. We housed groups of bats in acoustically isolated chambers and continuously monitored them with video cameras and microphones around-the-clock. Over the course of 75 days, we recorded tens of thousands of vocalizations, for many of which (~15,000) we were able to determine both the behavioral context as well as the identities of the emitter and the addressee. Importantly, our dataset covers the complete repertoire used by these bats over a period of 2.5 months, as we continuously recorded the vocalizations of the bats and their behavior.

One might expect most social interactions in a tightly packed group, such as a fruit bat colony, to be aggressive. Indeed, nearly all of the communication calls of the Egyptian fruit bat in the roost are emitted during aggressive pairwise interactions, involving squabbling over food or perching locations and protesting against mating attempts (Supplementary Videos S1,S2,S3,S4). These kinds of interactions, which are extremely common in social animals, are often grouped into a single “agonistic” behavioral category in bioacoustics studies. Moreover, in many bioacoustics studies, different calls are a-priori separated into categories by human-discernible acoustic features e.g. refs 14 and 37 (but see refs 10 and 38). Such an approach however, was impossible with our data (Fig. 1, note how aggressive calls emitted in different contexts seem and sound similar). We therefore adopted a machine-learning approach, which proved effective in recognizing human speakers^39^, and used it to evaluate the information potential of the spectral composition of these vocalizations. We were able to identify, with high accuracy, the emitters of the vocalizations, their specific aggressive contexts (e.g., squabbling over food), and to some extent, the addressees and the behavioral responses to the calls. Our study shows that bat vocalizations contain diverse types of information, already detectable in their spectral properties alone, and even among the seemingly similar aggressive contexts.

## Results

### Collected data

**Figure 1:**
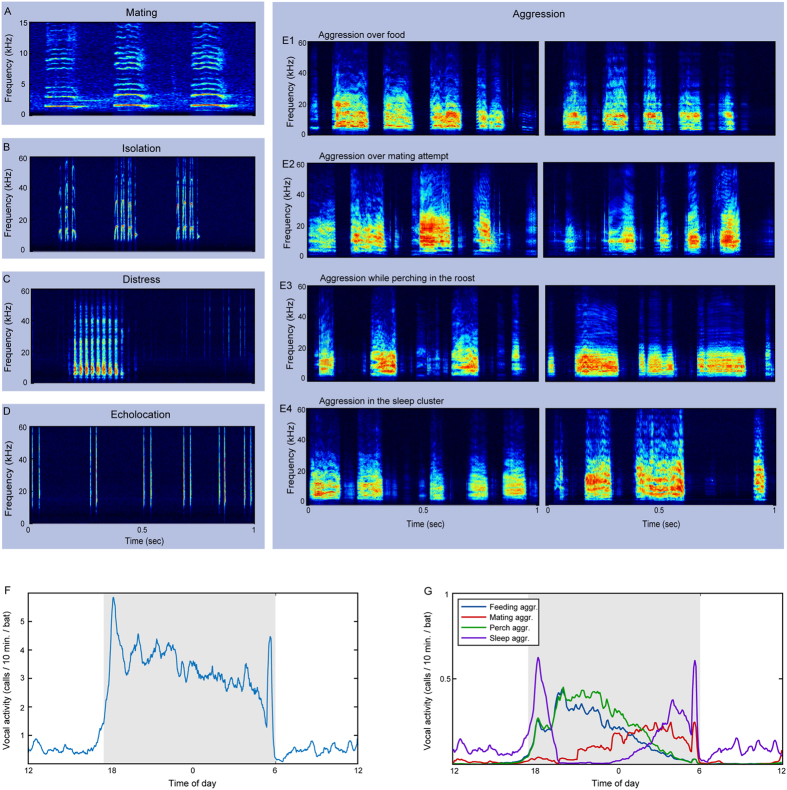
Distinct types of Egyptian fruit bat vocalizations. (**A–E**) Spectrograms of calls associated with different behaviors. Spectrograms show a time span of 1 sec. and a frequency band of 0–15 kHz (for A) and 0–60 kHz (for **B**–**E**). (**A**) Mating calls - produced by males during mating. (**B**) Isolation calls – produced by pups when left alone in the roost or when detached from their mothers. (**C**) Seclusion/distress calls – emitted mostly by distressed adults which are separated from others. (**D**) Echolocation clicks – lingual click-based sonar signals. (**E**) Aggression calls – the most common call type; produced during the many everyday squabbles between pairs of bats. These vocalizations are composed of sequences of broad-band multi-harmonic syllables (usually 1-20 syllables). Two examples for each aggression (aggr.) context: (E1) Feeding aggr., (E2) Mating aggr., (E3) Perch aggr., and (E4) Sleep aggr. (**F**) Occurrence of aggression calls along the day, presented as average calls/10-min. per bat. White/gray background indicates light/dark time of day. The peaks at the beginning/end of the night are associated with the disintegration/reintegration of the day-time sleeping cluster. (**G**) Occurrence of aggression calls in the four analyzed contexts (assessed according to the proportions of these contexts among annotated recordings).

75 days of continuous recordings of 22 bats (12 adults and 10 pups) yielded a dataset of 162,376 vocalizations, each consisting of a sequence of syllables (Fig. 1E). From synchronized videos we identified the emitter, addressee, context, and behavioral response. We included in the analysis 14,863 vocalizations of 7 adult females, for which we had enough data in the analyzed contexts (see Supplementary Tables S1–S3 and Materials and Methods). The spectral content of the vocalizations was represented by Mel-frequency cepstral coefficients (MFCC) which are the most common features used in human speech processing, and which were also found useful in the analysis of other animal vocalizations^10^,^40^ (and see Materials and Methods). A statistical model was built for each class in each attribute type, e.g. a model for each specific context, each individual bat, each combination of the two, etc. (see illustration in Supplementary Fig. S1, and Materials and Methods for full details). All results refer to classification of test data, which is data that were not used for designing the classifier (i.e. in cross-validation). Classification performance is reported as the balanced accuracy (BA), which is the number of correct classifications in each class, divided by the number of examples in each class, averaged over all classes. This measurement mitigates biases which could rise from unbalanced class sizes.

### Who addressed whom - recognizing the emitter and addressee

**Figure 2:**
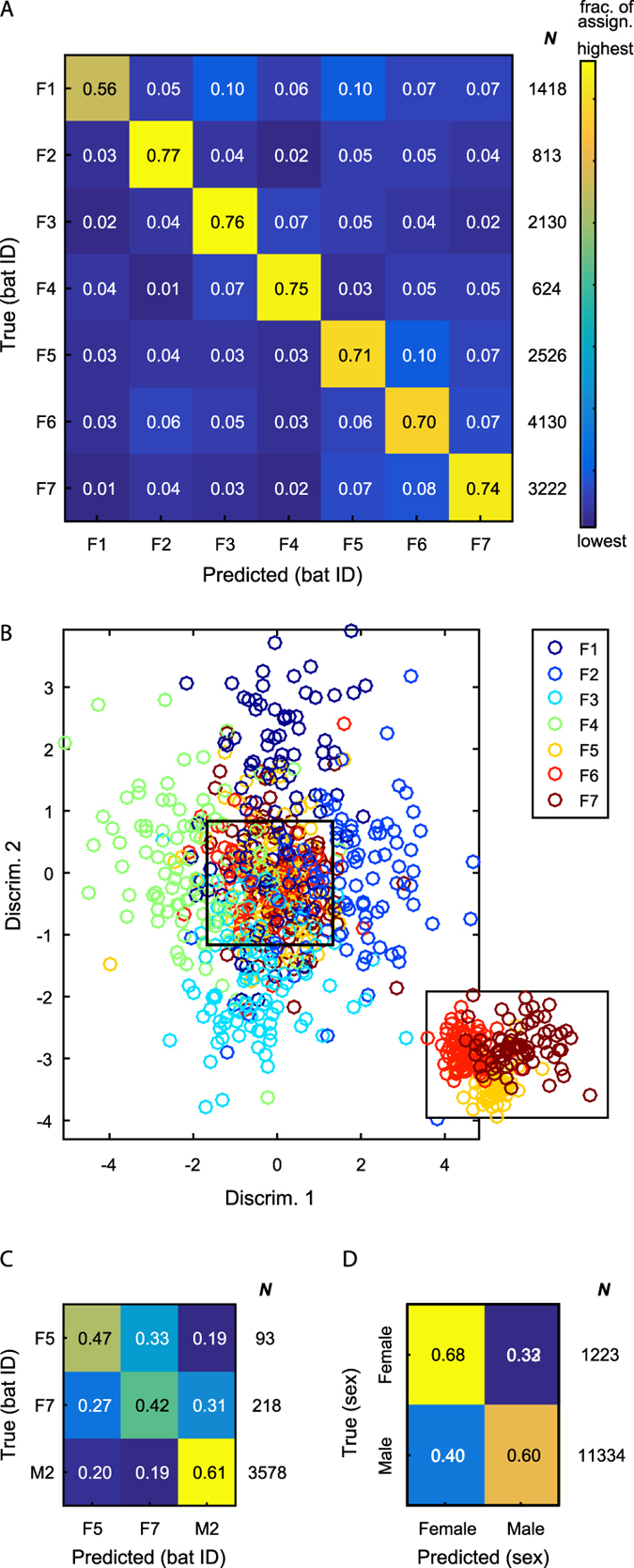
Emitter and addressee of the vocalizations. (**A**) A confusion matrix for the prediction of the emitting individuals. The number in each box indicates the fraction of each true class (row) assigned to each predicted class (column), i.e., each row sums to 1. The total number of vocalizations in each class (*N*) is indicated to the right of the matrix. Yellow – highest value in the matrix, Blue – lowest value. (BA = 71%, chance = 14%, p < 0.01) (**B**) 2D Projection of the vocalizations of different emitters (only test data) through the trained models: each vocalization is represented as a 7-dimensional vector – the score it received for each model, then LDA is applied (for visualization only) and the two first linear discriminators are used (see Materials and Methods). Inset: only emitters F5-F7 in an alternative projection. (**C**) A confusion matrix for the prediction of the addressees of vocalizations produced by bat F6 (BA = 50%, chance = 33%, p < 0.01). (**D**) A confusion matrix for the prediction of the vocalizations addressed to a male vs. those addressed to a female (BA = 64%, chance = 50%, p < 0.01).

The emitters of the vocalizations were clearly identified with a balanced-accuracy (BA) of 71% (where chance level was 14%, i.e. one out of seven, p < 0.01, permutation test, Fig. 2A,B, Supplementary Fig. S2), indicating that, potentially, a bat could acoustically recognize who is addressing it. We could also successfully recognize the emitter when incorporating a larger number of emitters (n = 15 individuals), using recordings of adult bats from a previous experiment (BA of 56% for 15 individuals, where chance level was 7%, Supplementary Fig. S3; see Materials and Methods), indicating that this result can be extended as data availability increases.

We then tested if we could use the same method in order to recognize the addressee (i.e. the target bat) of each vocalization. We found significant differences between vocalizations directed to different addressees. Figure 2C shows the results for the emitter for which we had the largest dataset (F6), with a BA of 50% (chance was 33%, p < 0.01, permutation test; see Supplementary Fig. S4 for other emitters. All but one emitter were significant). Note that we tested addressee classification for every emitter separately thus overcoming the risk of recognizing the emitter and not the addressee. Part of the information regarding the identity of the addressees resulted from the differences between calls directed to males and calls directed to females. Indeed, the addressee’s sex could be classified with a BA of 64% (chance was 50%, p < 0.01, permutation test; Fig. 2D). Nevertheless, when examining each emitter alone, the identity of specific female addressees (when males were excluded from the analysis) could be recovered above chance (for all but 1 emitter, Supplementary Fig. S4), revealing significant variations between calls directed at different recipients. This implies that an eavesdropping bat is theoretically able, to some extent at least, to identify if individual *A* is addressing individual *B* or individual *C*.

### What was the quarrel about - context identification

**Figure 3:**
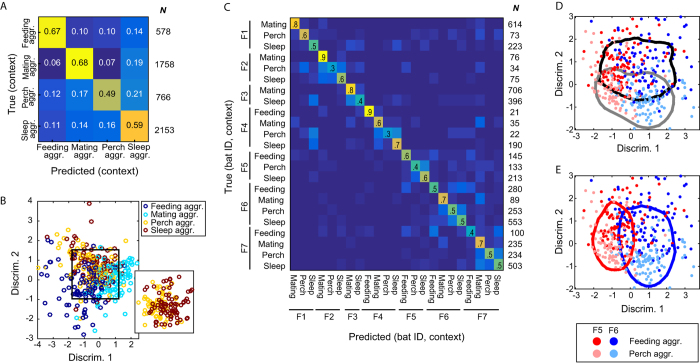
The context of the vocalizations. (**A**) A confusion matrix for the prediction of the context (BA = 61%, chance = 25%, p < 0.01). The number in each box indicates the fraction of each true class (row) assigned to each predicted class (column). The total number of vocalizations in each class (*N*) is indicated to the right of the matrix. (**B**) Projection of the different contexts through the trained models (see Materials and Methods). Inset: only contexts *perch aggr.* and *sleep aggr.* in another projection. (**C**) A confusion matrix for the prediction of the context and emitter together (BA = 57%, chance = 4%, p < 0.01; exact numbers for each cell are given in Supplementary Table S4). (**D** and **E**) Projection of two emitters – F5, F6, in two contexts – *feeding aggr.* and *perch aggr.* (**D** and **E**) depict the same plot: red – F5, blue – F6, dark color – *feeding aggr.*, light color – *perch aggr.* The black and grey lines in (**D**) portray the 0.3 isoline of the 2D histogram (with max. normalized to 1) of *feeding aggr.* and *perch aggr.*, respectively. The red and blue lines in (**E**) portray the 0.3 isoline of the 2D histogram (with max. normalized to 1) of F5 and F6, respectively.

We tested for differences between vocalizations produced in the four distinct, most common, aggressive contexts (see examples in Supplementary Videos S1,S2,S3,S4): *feeding aggression* (quarrelling over food), *mating aggression* (protest of females against males’ mating attempts), *perch aggression* (face-to-face aggressive display, where the two bats perch in close vicinity), and *sleep aggression* (squabbling in the sleeping cluster). Typically in these interactions, a bat aversively violates the personal space of another bat (e.g., by sniffing it or trying to grab food from its mouth), and the other bat’s reaction would include an acoustic protest. We could classify the behavioral context with a significant BA of 61% (chance was 25%, p < 0.01, permutation test), suggesting that an eavesdropper can potentially infer, fairly well, the context of the quarrel based on the vocalizations of the individuals involved (Fig. 3A,B). We controlled for biases that could arise from an unequal representation of the individuals in different contexts (e.g. individual *A* dominates sleeping interactions so that our classifier actually recognizes the emitter and not the context). To this end, we also classified the context of the calls for each individual emitter separately, and again received significant separations between contexts, and with even higher accuracies (an average BA of 75%; Supplementary Fig. S5). Furthermore, we were able to classify all context-emitter pairs (Fig. 3C). To illustrate the interplay between emitters and contexts we show the classification models constructed for two emitters (F5, F6) in two different contexts (*feeding aggr., perch aggr.*) in Fig. 3(D,E). This 2D projection demonstrates how emitter can be separated along one acoustic dimension while context varies along another one. We also controlled for the possibility that interactions with food (e.g. having food in one’s mouth) directly affected the acoustics of the feeding related vocalizations, by analyzing only feeding interactions for which we could verify that the emitter did not have food in its mouth. Again, the results were significant, with a BA of 64% (Supplementary Fig. S6).

### What was the outcome of the quarrel?

**Figure 4:**
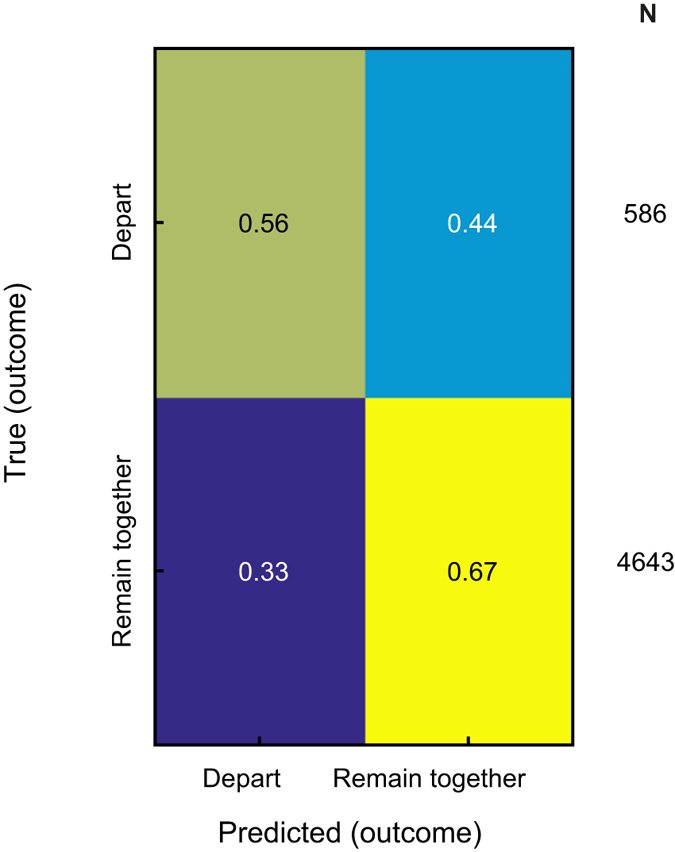
Predicting the behavioral outcome of the interaction. A confusion matrix for the prediction of the outcome of the vocal interactions. Two possible outcomes were defined: *Depart* and *Remain together* (BA = 62%, chance = 50%, p < 0.01). The number in each box indicates the fraction of each true class (row) assigned to each predicted class (column). The total number of vocalizations in each class (*N*) is indicated to the right of the matrix.

Following the realization that much information resides in the spectral content of bat aggressive calls, we examined whether we could predict the behavioral outcome of each aggressive vocal interaction. To this end, we defined two crude (but easily distinguishable) possible outcomes: departing (each bat goes its own way, or one stays in place while the other leaves), and remaining together (both bats remained close-by after the vocalization). Surprisingly, we could predict the outcome of a quarrel above chance level, with BA of 62% (chance is 50%, p < 0.01, permutation test) (Fig. 4). We further attempted to predict the outcome in more detail, namely to predict which individual of the interacting pair will stay and which will leave following the interaction. Again we could predict the detailed outcome significantly above chance level, with BA of 41% (chance is 25%, p < 0.01, permutation test; Fig. S7).

### Controlling for influence of emitter or context on the classifications of the addressee and the outcome

The classification accuracies of the addressee and the outcome were inferior to those of the emitter and context. To make sure that these classifications were not a byproduct of an unequal representation of the emitters or contexts, we performed a series of conservative tests classifying the addressee or the outcome for individual emitters in specific contexts. For addressee identification the classification accuracy was significantly better than chance for 12 out of the 14 tested cases (p < 0.01 in 11 cases, p = 0.04 in one case, n.s. in 2 cases: p = 0.08 and p = 0.09, permutation test; Supplementary Table S5). For the outcome classification the accuracy was significantly better than chance for 12 out of the 13 tested cases (p ≤ 0.01 in 8 cases, p ≤ 0.03 in 4 cases, n.s. in one case: p = 0.08, permutation test; Supplementary Table S6), and for the detailed outcome (which included the prediction of who stayed and who left) the accuracy was again significantly better than chance for all 12 tested cases (p ≤ 0.02, permutation test; Supplementary Table S7). We could also classify the context and the addressee’s sex for individual emitters. This classification was performed for two emitters for which we had enough data in all 8 combinations, i.e. male and female addressees in 4 contexts (emitter F5: BA = 40%, chance is 12.5%, p < 0.01; emitter F6: BA = 35%, chance is 12.5%, p < 0.01, permutation tests; Supplementary Fig. S8). These results indicate that, independently of the emitter and context, the vocalizations contain information about their addressee and about the outcome of the interaction.

## Discussion

We have shown that social vocalizations of fruit bats contain multilayered information. This vast information is available even in calls that would have previously been categorized as belonging to the same crude context of aggressive behavior, and which sound very similar to the human ear. We continuously recorded the bats, and assembled a dataset that represents the full vocal repertoire which was used during the experiment period. This very large dataset of vocal interactions included a careful categorization of the context and participants, and enabled us to uncover the information embedded in everyday aggressive vocalizations. While the natural function of this information is yet to be discovered, we define the encapsulated information as the *potential knowledge* that could be extracted from the acoustics alone (see ref. 41 for a discussion on the information in animal signals). To a listener bat, the spectral composition alone already holds information about the identity of the emitter of a specific call and its specific aggressive context. Furthermore, the outcome of the interaction can be predicted above chance level by listening. Interestingly, the spectral composition also carries information about the addressee of a vocalization. Even if the acoustic differences were merely a result of the emotional state of the animal, an eavesdropper (or an addressee) could potentially use them to extract valuable information^42^.

It is important to note that we used one set of acoustic features for classification. However, many other multi-dimensional spectro-temporal representations can be tested. The bat’s brain could thus be using some other representation that encapsulates much more information regarding different social aspects. The bat may be able to classify the context of an interaction with higher confidence, based on some acoustic feature which it evolved to use and is yet to be determined. Our analysis is thus probably only a lower bound on what a bat is capable of extracting from aggressive social vocalizations. For example, we did not include any temporal information in our analysis. Temporal features (e.g. amplitude and frequency modulations) exhibit large variability among bat vocalizations, and are known to contribute much to human speech recognition (e.g. ref. 43). Furthermore, a two-step classification process improved the accuracy. For example, the accuracy of identifying the context of a vocalization increased to 75% when the emitter was classified first, as opposed to 61% when the emitter was unknown.

**Figure 5:**
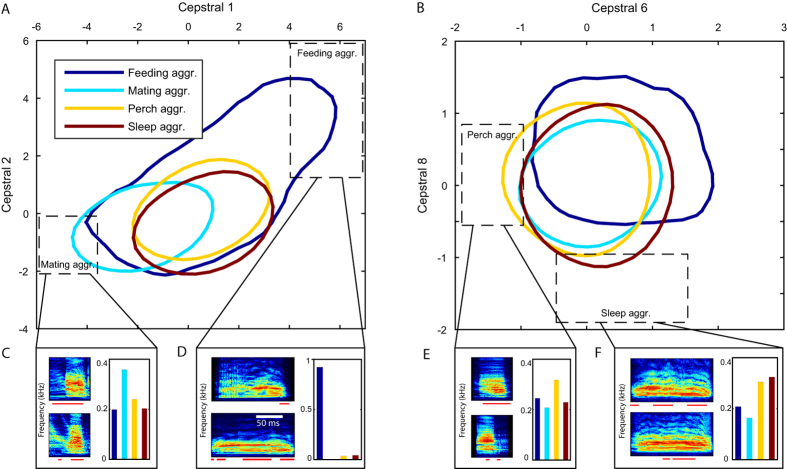
Acoustic features conveying the information. An example of the distribution of two pairs of acoustic features in the analyzed contexts. Colored lines mark the 0.5 isoline of the 2D histograms (with max. normalized to 1). (**A**) Mel-cepstral coefficients 1 and 2. (**B**) Mel-cepstral coefficients 6 and 8. In both cases, the distributions mostly overlap each other. The least overlapping regions are marked with dashed rectangles. (**C–F**) For each such region: the fraction of each context, out of the vocalizations which contains values inside the marked rectangle, is shown in the bar plot. Spectrograms of two syllables from the prevalent context in each region are presented, i.e. (**C**) *mating aggr.*, (**D**) *feeding aggr.* (**E**) *perch aggr.* (**F**) *sleep aggr.* Red bars below the spectrograms mark the regions which contains values inside the marked rectangle. For all spectrograms: frequency axis is 0–40 kHz, time scale as indicated in (**D**). To avoid any possible biases in this example, the displayed distributions are taken only from one recording channel (the one with the largest yield, channel 3, cage 2), all of the spectrograms are from vocalizations produced by the same individual (F7), and in each pair of spectrograms - each was taken from a different day. In total 64 cepstral coefficients were used to describe vocalizations, allowing separation of classes among many more dimensions.

While our results demonstrate that the spectral content of fruit bat vocalizations encapsulates valuable information, it is not straightforward to pinpoint a specific acoustic feature which uniquely defines one vocal class. When examining the four analyzed behavioral contexts, for instance, the distribution of the cepstral coefficients (which were used for classification) reveals a widespread overlap between different contexts (Fig. 5). Yet, each context encompasses specific regions of extreme values which are more unique to it than to the other contexts (Fig. 5A,B, dashed rectangles). Since each vocalization contains various values for each feature (changing along and between the syllables), it is sufficient that the acoustics of a small portion of the vocalization are outside the overlapping region for its context to be correctly classified. This type of inference, which is based on a distinguishable part of a sequence, may be analogous to classifying a conversation as food-related if the word “banana” appears somewhere in the sentence. Figure 5 depicts examples of syllables that contain such extreme values of cepstral coefficients in different contexts.

One of our surprising results was that vocalizations contain some information about the identity of their addressee. Some studies have suggested the existence of addressee specific calls in dolphins^44^; others have demonstrated how audience composition may affect primate vocalizations^45^. Our finding might be akin to a human speaker who uses varying intonation towards different listeners (e.g. male vs. female addressees) while using the same words.

The importance of vocal communication increases when vision is limited (e.g. in dense forests or underwater), thus it is reasonable to assume that the vocalizations of a social mammal, which roosts in dark caves, will evolve to convey elaborate information about the interactions between individuals. Most of this species’ vocalizations are emitted during aggressive encounters that include squabbling over food, sleeping spots, or other resources. Importantly, these vocalizations are directed at a specific addressee - the interacting peer (as is the case of human speech), rather than broadcast, as is the case of birdsongs, many types of alarm calls, and human non-linguistic vocal behaviors such as laughter, crying, or screams. Such mundane calls, which accompany routine behaviors, account for the great majority of fruit bat communication and govern the vocal communication of many species. However, these calls are often grouped into one category in acoustic studies due to the difficulty of cataloguing them in detail, both acoustically and behaviorally. Inspired by the behavioral usage of human language, we believe that it is extremely beneficial to delve into the everyday chitchat among animals, when attempting to track the route of language evolution.

## Materials and Methods

### Animal retrieval and care

Adult bats (*Rousettus aegyptiacus*) were captured in a natural roost near Herzliya, Israel. This roost is regularly inhabited by a colony of 5,000 to 10,000 bats. The bats were kept in acoustic chambers, large enough to allow flight, and fed with a variety of local fruit. All experiments were reviewed and approved by the Animal Care Committee of Tel Aviv University (Number L-13-016), and were performed in accordance with its regulations and guidelines regarding the care and use of animals for experimental procedures. The use of bats was approved by the Israeli National Park Authority.

### Bat housing and monitoring

The bats were housed in 2 identical cages (acoustic chambers; for illustrations refer to ref. 36), with 6 females, 5 pups, and 1 male in cage 1, and 4 females, 4 pups, 1 male, and 1 young (of unknown sex) in cage 2. The cages were continuously monitored for 75 days, with IR-sensitive cameras and omnidirectional electret ultrasound microphones (Avisoft-Bioacoustics Knowles FG-O; 2 microphones in each cage). Audio was sampled using Avisoft-Bioacoustics UltraSoundGate 1216HA/D converter with a sampling rate of 250 kHz. The chambers were acoustically isolated and their walls were covered with foam to diminish echoes. Raw audio recordings were automatically segmented and filtered for noises and echolocation clicks, leaving only bat social communication calls (see ref. 36 for details of this process). Video was synchronized to the audio, resulting in a short movie accompanying each audio recording. Videos were then analyzed by trained students, who identified the circumstances of each call (emitter, addressee, context, and behavioral response, see details below). The bats were individually marked using a collar with a reflective disc. The observers were cross validated during their training to ensure qualified annotations. An emitter bat was recognized by its mouth movements, and 2–3 cameras could be used to verify a distinct assignment. If there was any doubt regarding the emitter’s identity we excluded the vocalization from the analysis. This conservative approach is the main cause for the exclusion of almost 90% of the vocalizations from our analysis. There was a negligible number of events when two bats vocalized together (or shortly after each other). These events could be easily distinguished from the spectrograms.

### Classification tasks

We managed to annotate 19,021 calls with all of the required details for classification. We then only used individuals for which we had enough vocalizations in at least 3 of the tested contexts (at least 15 per context). The analyzed data hence consists of 14,863 calls produced by 7 adult females (F1–F7). We classified the emitter of the vocalization among these 7 females (the males produced much less vocalizations, hence were not used in this study). For extending the emitter recognition to a larger number of individuals, we used all bats, including adults which were previously recorded in the same setup, excluding pups and those with less than 400 recorded vocalizations, ending up with 15 individuals (Supplementary Fig. S3). Four aggressive contexts were included in the analysis: (1) *Feeding aggression* – interactions during feeding or in close proximity to the food; (2) *Mating aggression* – produced by females in protest to males’ mating attempts; (3) *Perch aggression* – emitted when two bats who perched close to each other, confronted one another, displaying aggressive acts, accompanied by rapid movements of the wings, but with no close contact; and (4) *Sleep aggression* – squabbling over locations, or other aggression, in the day-time sleeping cluster. For the emitter and addressee classification we also included vocalizations for which the context was not conclusive (“*General*” in Supplementary Table S1). In this *General* (unidentified) aggression context the interacting bats are usually 10–20 *cm* apart, while in the other contexts they were *ca*. 0–10 *cm* apart. The *Mating aggression* context was not used in the addressee classification task, as these vocalizations were exclusively directed toward the male (hence identifying the addressee in this case could result from solely identifying the context). In the prediction of the addressees of the vocalizations we used all addressees with at least 20 calls addressed to them. The outcome of a vocal interaction was defined as one of two options: (1) *Depart* – the two bats split after the interaction, where either both went their own way, or one of them left and the other stayed in place, (2) *Remain together* – the two interacting bats stayed in the same position (in close proximity) after the interaction ended. In controlling for emitter/context influence on addressee/outcome classifications (i.e. vocalizations in specific context by individual emitters) we allowed classes (addressees or outcomes) with at least 10 calls, in order to extend the coverage of different cases.

### Feature extraction for classification

Egyptian fruit bat vocalizations consist of multisyllabic sequences, with short periods of silence between the syllables (Fig. 1E, Supplementary Fig. S1). Each vocalization was first automatically segmented (as described in ref. 36), retrieving only the voiced segments (see “voiced” and “unvoiced” bars in Supplementary Fig. S1). Then, a sliding window of 20 *ms* (with an overlap of 19 *ms* between consecutive windows) was used to extract 64 Mel-frequency cepstral coefficients (MFCC) from each window. The MFCC assumes a logarithmic pitch scale which is typical for mammals (including bats)^46^. The mel-scale was originally tuned for human perception. However, as we did not intend to mimic the bat’s auditory system precisely, but only to test for available information and as there is no equivalent bat scale, we chose to use it. The feature vectors retrieved from all segments were joined to one set of several 64-dimentional feature vectors representing the vocalization (Supplementary Fig. S1). The MFCCs were normalized by subtracting their mean for every recording channel (2 channels in each cage) as is commonly done to reduce (recording) channel biases.

### Classification algorithm and cross validation

The GMM-UBM algorithm was used for classification (following^39^, which used it for human speaker recognition). In short, given a labeled training set of vocalizations, for each class (e.g. emitter “F1”, the context “*Feeding aggr.*”, etc.) all sets of feature vectors from all vocalizations of this class are pooled together into one mega-set. This mega-set is then modeled by a Gaussian mixture model (GMM) of 16 Gaussian components. However, instead of directly fitting the GMM onto the data, the GMM parameters are assessed using an adaptive method, based on a universal background model (UBM) (see details of the procedure in Supplementary Fig. S1 and Supplementary Methods). The UBM is a GMM fitted to another set of data, which was not used for training or testing. To this end, we employed the data which was not part of the analysis due to lack of detailed annotations. We sampled a random sample of syllables from all of the vocalizations for which the identity of the pair was known but the role of each individual was not certain (i.e. who addressed whom). This sample constituted the background set of 3900 syllables, and its corresponding UBM was used for all of the classifications. A test sample, i.e. a vocalization unseen by the model training algorithm, then received a score for each possible class (e.g., each context). The score was computed as the ratio between the likelihood that the sample was drawn from the specified class (computed using the learned GMM) and the likelihood that the sample was drawn from the UBM. This process results with each sample in the test set having a score for each possible class, and the class with the maximum score is chosen as the prediction for this sample. Due to small sample size in some classes, in a few classification tasks, we adopted a leave-one-out approach for cross-validation (for all tasks): Iteratively, over the entire set, each vocalization at a time is left outside of the training set and then given a prediction by the trained model (which means that every prediction is made on a vocalization unseen by the training algorithm). The success of the classification was measured using the balanced accuracy (BA): First, the confusion matrix is normalized by each class size (i.e., the sum of each row is 1, and the diagonal holds the fraction of correct predictions in each class), then, the BA is the average of the confusion matrix diagonal. To estimate a p-value for each success rate we ran permutation tests, where we permuted the labels of the original set (e.g., we mixed the contexts). 100 permutations were used in each test. To exclude any influence of context-dependent background noises on the classification success, we verified that our results could not be replicated by classifying non-voiced recordings from the analyzed contexts.

### 2D projections for visualization

Each vocalization is represented in our data as a set of 64-dimensional vectors, where this set is of varying size, depending on the duration of the vocalization. In order to illustrate the interplay between the acoustics of different classes we projected each vocalization onto a 2-dimensional plane (Figs 2B and 3B,D,E). To this end, we assigned each vocalization a new “feature” vector containing the scores it received from our algorithm for each class. Thus, each vocalization was represented by a single *C*-dimensional vector (where *C* is the number of classes in the classification task, e.g. 7 for emitter classification). For visualization, we now used the first 2 dimensions of a linear discriminant analysis applied to this new set of *C*-dimensional vectors. This process can be viewed as a type of “multi-dimensional scaling”: from a variable number of dimensions (each vocalization was described by a different number of 64-dimensional vectors), through the lens of our models, onto a 2 dimensional plane. Importantly, this process is done on scores received when the vocalizations were in the test set, i.e. unseen by the training algorithm.

## Additional Information

**How to cite this article**: Prat, Y. *et al*. Everyday bat vocalizations contain information about emitter, addressee, context, and behavior. *Sci. Rep.* **6**, 39419; doi: 10.1038/srep39419 (2016).

**Publisher's note:** Springer Nature remains neutral with regard to jurisdictional claims in published maps and institutional affiliations.

## Supplementary Material

Supplementary Video S1

Supplementary Video S2

Supplementary Video S3

Supplementary Video S4

Supplementary Information
