# Supplementary material for: Everyday bat vocalizations contain information about emitter, addressee, context, and behavior
Source: Sci Rep. 2016 Dec 22;6:39419. doi: 10.1038/srep39419 (PMC5178335; doi:10.1038/srep39419)
Supplement: Supplementary Information [file srep39419-s5.pdf]

Everyday bat vocalizations contain information about emitter, addressee, context, and behavior

Yosef Prat[1], Mor Taub[1], and Yossi Yovel[1]*

Author affiliations: [1]Department of Zoology, Faculty of Life sciences, Tel Aviv University, Tel Aviv, Israel.

***Corresponding author:** Yossi Yovel, Department of Zoology, Faculty of Life sciences, Tel Aviv University, Tel Aviv, 69978, Israel. E-mail: yossiyovel@hotmail.com

Supplementary Information

Supplementary tables

Emitter (Bat ID)	Feeding aggr.	Mating aggr.	Perch aggr.	Sleep aggr.	General*	Total
F1	7	614	73	223	501	1418
F2	19	76	34	75	609	813
F3	6	706	17	396	1005	2130
F4	21	35	22	190	356	624
F5	145	3	133	213	2032	2526
F6	280	89	253	553	2955	4130
F7	100	235	234	503	2150	3222
**Total** (included)	578	1758	766	2153	9608	14863
P4	12	0	4	17	74	107
P5	0	0	0	4	14	18
P1	1	0	1	31	75	108
P6	1	0	0	1	36	38
P7	2	0	14	25	69	110
P2	5	4	16	80	211	316
M1	1	1	1	6	77	86
F9	96	5	13	115	892	1121
P3	9	4	2	27	205	247
P8	2	0	2	29	82	115
F10	0	0	1	52	62	115
F8	5	0	4	252	338	599
U1	75	0	3	53	648	779
P9	3	0	1	39	153	196
M2	11	0	17	7	168	203
Unknown	772	444	559	1873	9212	12860
**Total** (not included)	995	458	638	2611	12316	17018

Table S1. Number of annotated vocalizations by context. The upper section presents vocalizations which were included in the analysis. Individuals which had less than 15 vocalizations in more than one context were excluded. The lower section presents vocalizations of these individuals. Bat ID: Fx – Female, Mx – Male, Px – Pup, Ux – Young adult of unknown sex.

* Vocalizations of "*General*" context were included only in the emitter and addressee classifications.

Emitter (Bat ID)	Depart	Remain together	Unknown	Total (known)
F1	15	900	2	922
F2	30	174	0	208
F3	29	1079	17	1113
F4	11	255	2	269
F5	133	359	2	495
F6	181	993	1	1193
F7	187	883	2	1074
**Total** (included)	586	4643	(26)*	5274
P4	13	19	1	32
P5	2	2	0	4
P1	9	24	0	33
P6	0	2	0	2
P7	14	27	0	41
P2	33	72	0	105
M1	0	8	1	8
F9	112	116	1	228
P3	12	30	0	42
P8	7	26	0	33
F10	0	53	0	53
F8	14	247	0	265
U1	43	88	0	131
P9	9	34	0	43
M2	8	27	0	36
Unknown	654	2937	57	3591
**Total** (not included)	930	3712	60	4647

Table S2. Number of annotated vocalizations by outcome. The upper section presents vocalizations which were included in the analysis. Individuals which have less than 15 vocalizations in more than one context (see Table S1) were excluded. The lower section presents vocalizations of these individuals. Only vocalizations in which the outcome could be clearly defined were included.
* Unknown outcomes were not included.

Emitter	Addressee											
Bat ID	F1	F2	F3	F4	F8	M1	P1	P4	P6	P8	F10	P9	Unknown
F1	0	228	69	47	109	321	0	1	0	0	4	0	25
F2	14	0	4	4	2	675	0	1	2	0	1	1	33
F3	3	15	0	12	6	1306	32	1	3	1	2	1	42
F4	7	33	12	0	10	499	2	0	2	0	3	0	21
**Total**	24	276	85	63	127	2801	34	3	7	1	10	2	121

Bat ID	F5	F6	F7	F9	U1	M2	P1	P2	P3	P7	Unknown
F5	0	28	160	20	12	2224	0	11	0	5	63
F6	93	0	218	11	10	3578	0	16	6	3	106
F7	43	47	0	18	4	2731	0	2	15	5	122
**Total**	136	75	378	49	26	8533	0	29	21	13	291

Table S3. Number of annotated vocalizations by addressee. The upper section presents vocalizations from cage 1 and the lower section presents vocalizations from cage 2. Only addressees with more than 20 vocalizations addressed to them were included in each classification task. The majority of the vocalizations are produced by females and addressed to males. The table does not include *mating aggr.* context in which the addressee is always the male, hence classifying the addressee may be equivalent to predicting the context in these cases.

Predicted:		F1			F2			F3		F4				F5			F6				F7				*N*
True:		MA	PA	SA	MA	PA	SA	MA	SA	FA	MA	PA	SA	FA	PA	SA	FA	MA	PA	SA	FA	MA	PA	SA	
F1	MA	**0.76**	0.03	0.00	0.05	0.01	0.00	0.00	0.00	0.00	0.01	0.00	0.00	0.01	0.01	0.00	0.00	0.01	0.00	0.01	0.03	0.04	0.01	0.00	614
	PA	0.04	**0.64**	0.03	0.01	0.01	0.00	0.00	0.01	0.00	0.00	0.00	0.00	0.00	0.03	0.01	0.03	0.00	0.04	0.01	0.00	0.07	0.00	0.05	73
	SA	0.02	0.04	**0.47**	0.01	0.00	0.02	0.08	0.06	0.01	0.00	0.02	0.07	0.02	0.00	0.04	0.01	0.01	0.00	0.04	0.01	0.03	0.00	0.02	223
F2	MA	0.01	0.00	0.00	**0.87**	0.01	0.05	0.00	0.01	0.00	0.00	0.01	0.00	0.00	0.00	0.00	0.00	0.01	0.00	0.00	0.00	0.01	0.00	0.00	76
	PA	0.03	0.12	0.03	0.18	**0.29**	0.15	0.03	0.06	0.00	0.00	0.00	0.00	0.06	0.00	0.00	0.00	0.00	0.00	0.00	0.00	0.06	0.00	0.00	34
	SA	0.00	0.03	0.05	0.12	0.11	**0.56**	0.00	0.00	0.00	0.00	0.00	0.00	0.01	0.01	0.00	0.04	0.00	0.00	0.01	0.03	0.01	0.00	0.01	75
F3	MA	0.00	0.00	0.00	0.00	0.01	0.03	**0.80**	0.07	0.00	0.01	0.00	0.01	0.01	0.00	0.01	0.00	0.01	0.01	0.01	0.01	0.01	0.00	0.00	706
	SA	0.01	0.02	0.14	0.01	0.00	0.02	0.18	**0.39**	0.01	0.01	0.02	0.05	0.03	0.01	0.01	0.01	0.02	0.02	0.02	0.02	0.03	0.01	0.01	396
F4	FA	0.00	0.00	0.00	0.05	0.00	0.00	0.00	0.05	**0.86**	0.05	0.00	0.00	0.00	0.00	0.00	0.00	0.00	0.00	0.00	0.00	0.00	0.00	0.00	21
	MA	0.06	0.00	0.00	0.00	0.00	0.00	0.06	0.00	0.03	**0.63**	0.03	0.03	0.00	0.03	0.00	0.00	0.00	0.00	0.00	0.00	0.11	0.00	0.03	35
	PA	0.00	0.05	0.18	0.00	0.00	0.00	0.00	0.00	0.00	0.14	**0.27**	0.23	0.00	0.00	0.00	0.00	0.00	0.05	0.05	0.05	0.00	0.00	0.00	22
	SA	0.00	0.00	0.11	0.00	0.00	0.00	0.03	0.02	0.00	0.09	0.03	**0.66**	0.00	0.01	0.01	0.01	0.01	0.01	0.00	0.00	0.03	0.00	0.00	190
F5	FA	0.03	0.01	0.00	0.03	0.01	0.01	0.00	0.01	0.02	0.00	0.02	0.01	**0.56**	0.03	0.03	0.08	0.01	0.02	0.02	0.07	0.01	0.01	0.01	145
	PA	0.01	0.00	0.00	0.02	0.02	0.00	0.00	0.01	0.00	0.02	0.00	0.00	0.04	**0.44**	0.21	0.02	0.01	0.05	0.02	0.04	0.05	0.05	0.02	133
	SA	0.00	0.01	0.03	0.00	0.00	0.00	0.00	0.01	0.01	0.00	0.00	0.01	0.04	0.11	**0.59**	0.02	0.00	0.01	0.03	0.01	0.06	0.00	0.03	213
F6	FA	0.01	0.01	0.00	0.00	0.00	0.01	0.01	0.01	0.01	0.00	0.00	0.00	0.09	0.02	0.00	**0.46**	0.02	0.09	0.12	0.09	0.01	0.01	0.02	280
	MA	0.01	0.00	0.00	0.02	0.00	0.00	0.00	0.02	0.00	0.00	0.00	0.02	0.01	0.01	0.02	0.00	**0.66**	0.03	0.01	0.00	0.12	0.02	0.02	89
	PA	0.00	0.00	0.01	0.01	0.02	0.01	0.00	0.00	0.00	0.00	0.01	0.00	0.01	0.02	0.00	0.04	0.12	**0.51**	0.15	0.02	0.03	0.02	0.00	253
	SA	0.00	0.00	0.01	0.02	0.00	0.02	0.00	0.01	0.00	0.00	0.00	0.01	0.01	0.02	0.03	0.06	0.06	0.17	**0.50**	0.01	0.04	0.01	0.03	553
F7	FA	0.00	0.00	0.00	0.03	0.00	0.04	0.00	0.02	0.03	0.01	0.00	0.00	0.07	0.03	0.01	0.15	0.00	0.00	0.05	**0.35**	0.05	0.04	0.12	100
	MA	0.01	0.00	0.00	0.00	0.00	0.00	0.02	0.00	0.01	0.01	0.00	0.01	0.01	0.01	0.02	0.01	0.07	0.00	0.00	0.03	**0.68**	0.03	0.06	235
	PA	0.00	0.00	0.00	0.00	0.01	0.00	0.00	0.00	0.00	0.00	0.01	0.00	0.02	0.01	0.01	0.02	0.02	0.01	0.01	0.03	0.11	**0.53**	0.18	234
	SA	0.00	0.00	0.00	0.00	0.01	0.01	0.00	0.02	0.00	0.00	0.00	0.00	0.00	0.01	0.04	0.02	0.01	0.01	0.05	0.02	0.17	0.11	**0.50**	503

Table S4. A confusion matrix for the prediction of the context and emitter together (numbers for Fig. 3C). FA – Feeding Aggression, MA – Mating Aggression, PA – Perch Aggression, SA – Sleep Aggression.

Emitter (Bat ID)	Context	# Addressees	Chance-level	Balanced-Accuracy	p-value (permutation test)
F5	Feeding aggr.	2	50%	61%	0.09
F6	Feeding aggr.	3	33%	51%	**< 0.01**
F1	Perch aggr.	2	50%	85%	**< 0.01**
F5	Perch aggr.	2	50%	74%	**< 0.01**
F6	Perch aggr.	2	50%	55%	0.08
F1	Sleep aggr.	4	25%	50%	**< 0.01**
F5	Sleep aggr.	2	50%	68%	**< 0.01**
F6	Sleep aggr.	2	50%	68%	**< 0.01**
F1	General aggr.	5	20%	34%	**< 0.01**
F3	General aggr.	2	50%	73%	**< 0.01**
F4	General aggr.	2	50%	61%	**0.04**
F5	General aggr.	4	25%	51%	**<0.01**
F6	General aggr.	4	25%	39%	**< 0.01**
F7	General aggr.	4	25%	53%	**< 0.01**

Table S5. Control for emitter and context influence on addressee prediction. Each line represents a classification test for an individual emitter in a specific context. We only used cases for which there were at least 10 vocalizations addressed to each of at least 2 individuals. Permutation test of 100 iterations were performed for each classification. Significant p-values are in bold.

Emitter (Bat ID)	Context	# Outcomes	Chance-level	Balanced-Accuracy	p-value (permutation test)
F5	Feeding aggr.	2	50%	72%	**< 0.01**
F6	Feeding aggr.	2	50%	67%	**< 0.01**
F7	Feeding aggr.	2	50%	64%	**0.02**
F2	Perch aggr.	2	50%	69%	**0.03**
F5	Perch aggr.	2	50%	67%	**< 0.01**
F6	Perch aggr.	2	50%	56%	**0.02**
F7	Perch aggr.	2	50%	59%	**0.01**
F1	Sleep aggr.	2	50%	83%	**< 0.01**
F2	Sleep aggr.	2	50%	75%	**< 0.01**
F3	Sleep aggr.	2	50%	60%	**0.01**
F5	Sleep aggr.	2	50%	67%	**< 0.01**
F6	Sleep aggr.	2	50%	59%	**0.02**
F7	Sleep aggr.	2	50%	55%	0.08

Table S6. Control for emitter and context influence on outcome prediction. Each line represents a classification test for an individual emitter in a specific context. We only used cases for which there were at least 10 vocalizations resulting in each of the 2 possible outcomes. Permutation test of 100 iterations were performed for each classification. Significant p-values are in bold.

Emitter (Bat ID)	Context	# Outcomes	Chance-level	Balanced-Accuracy	p-value (permutation test)
F5	Feeding aggr.	3	33%	60%	**<0.01**
F6	Feeding aggr.	3	33%	45%	**<0.01**
F7	Feeding aggr.	3	33%	52%	**<0.01**
F5	Perch aggr.	2	50%	77%	**<0.01**
F6	Perch aggr.	4	25%	41%	**<0.01**
F7	Perch aggr.	3	33%	45%	**0.02**
F1	Sleep aggr.	2	50%	82%	**<0.01**
F2	Sleep aggr.	2	50%	75%	**<0.01**
F3	Sleep aggr.	2	50%	59%	**0.02**
F5	Sleep aggr.	3	33%	65%	**<0.01**
F6	Sleep aggr.	3	33%	39%	**0.01**
F7	Sleep aggr.	3	33%	40%	**0.01**

Table S7. Control for emitter and context influence on detailed outcome prediction. Each line represents a classification test for an individual emitter in a specific context. We only used cases for which there were at least 10 vocalizations resulting in at least 2 of the 4 possible outcomes. Permutation test of 100 iterations were performed for each classification. Significant p-values are in bold.

Supplementary figures

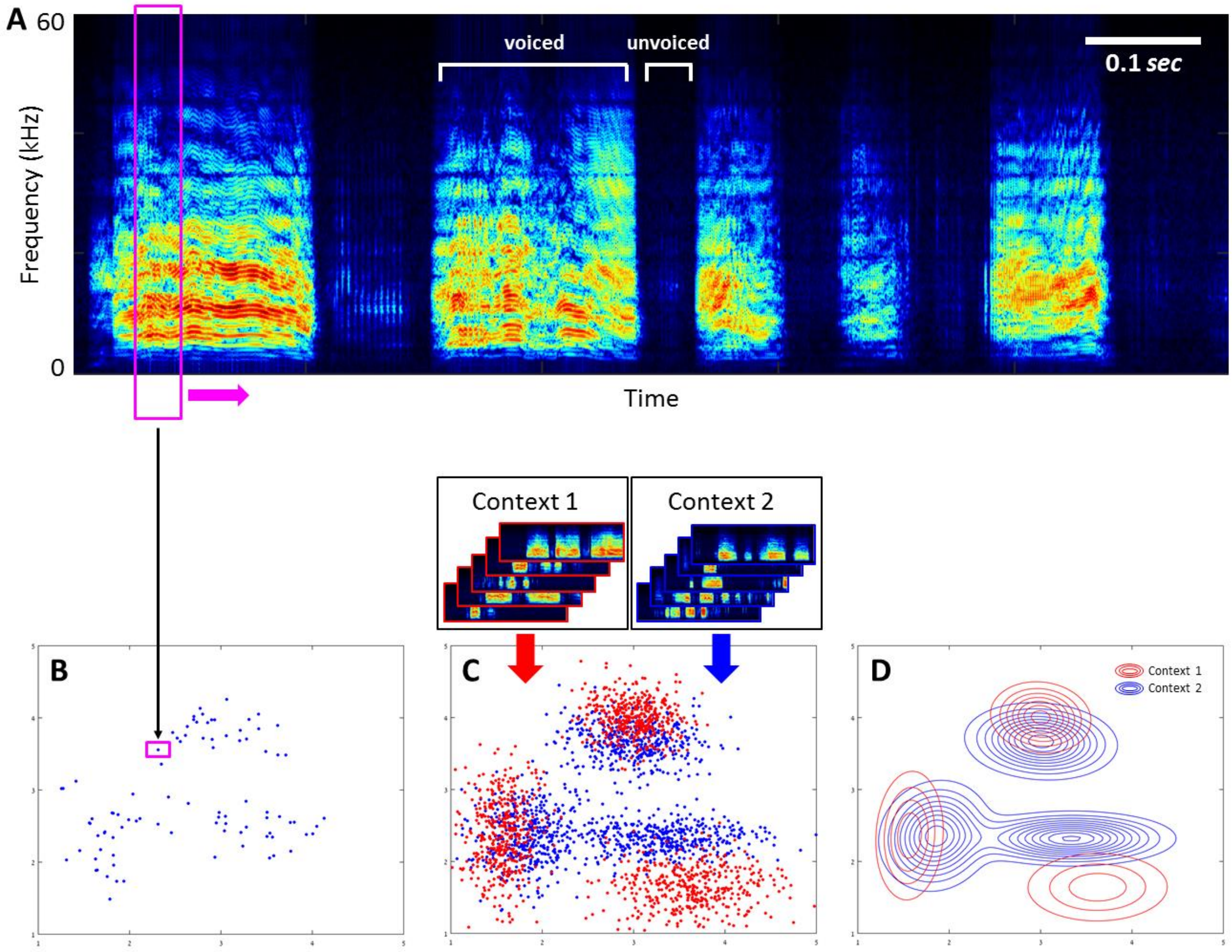

Figure S1. Flow chart of the analysis process. (**A**) Example of a spectrogram of a single Egyptian fruit bat vocalization, consisting of a bout of short syllables (marked as voiced) with low fundamental and rich harmonic content. Each vocalization is first automatically segmented retrieving the voiced segments, and removing the unvoiced parts (see Materials and Methods). (**B**) Spectral features (MFCC) were calculated using a sliding window resulting in a series of multi-dimensional vectors representing each vocalization (one vector for each window; the entire cloud of blue dots represents the vocalization in (A); dots are illustrative only). (**C**) All vocalizations of each class (e.g. context) were pooled together and a GMM was fitted to the distribution of their MFCCs (in an adaptive manner, see Materials and Methods and SI Methods). (**D**) The fitted models could then be used to predict the class of an unseen data.

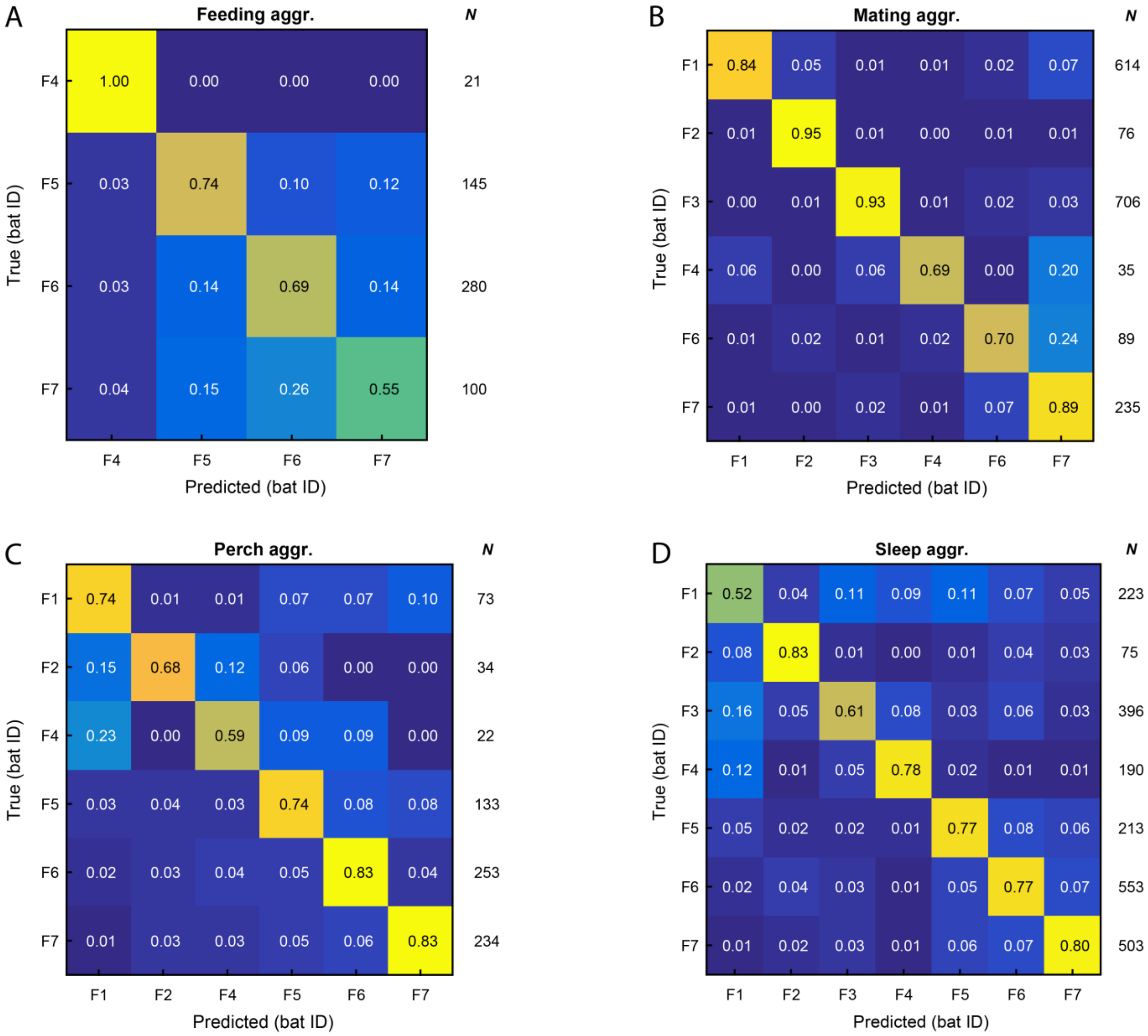

Figure S2. Emitter identification in specific contexts. Confusion matrices for the prediction of the emitting individuals. The number in each box indicates the fraction of each true class (row) assigned to each predicted class (column), i.e., each row sums to 1. The total number of vocalizations in each class (*N*) is indicated to the right of the matrix. Yellow – highest value in the matrix, Blue – lowest value. (**A**) Vocalizations produced in *feeding aggr.* context (BA=75%, chance=25%, p<0.01). (**B**) Vocalizations produced in *mating aggr.* context (BA=83%, chance=17%, p<0.01). (**C**) Vocalizations produced in *perch aggr.* context (BA=73%, chance=17%, p<0.01). (**D**) Vocalizations produced in *sleep aggr.* context (BA=73%, chance=14%, p<0.01).

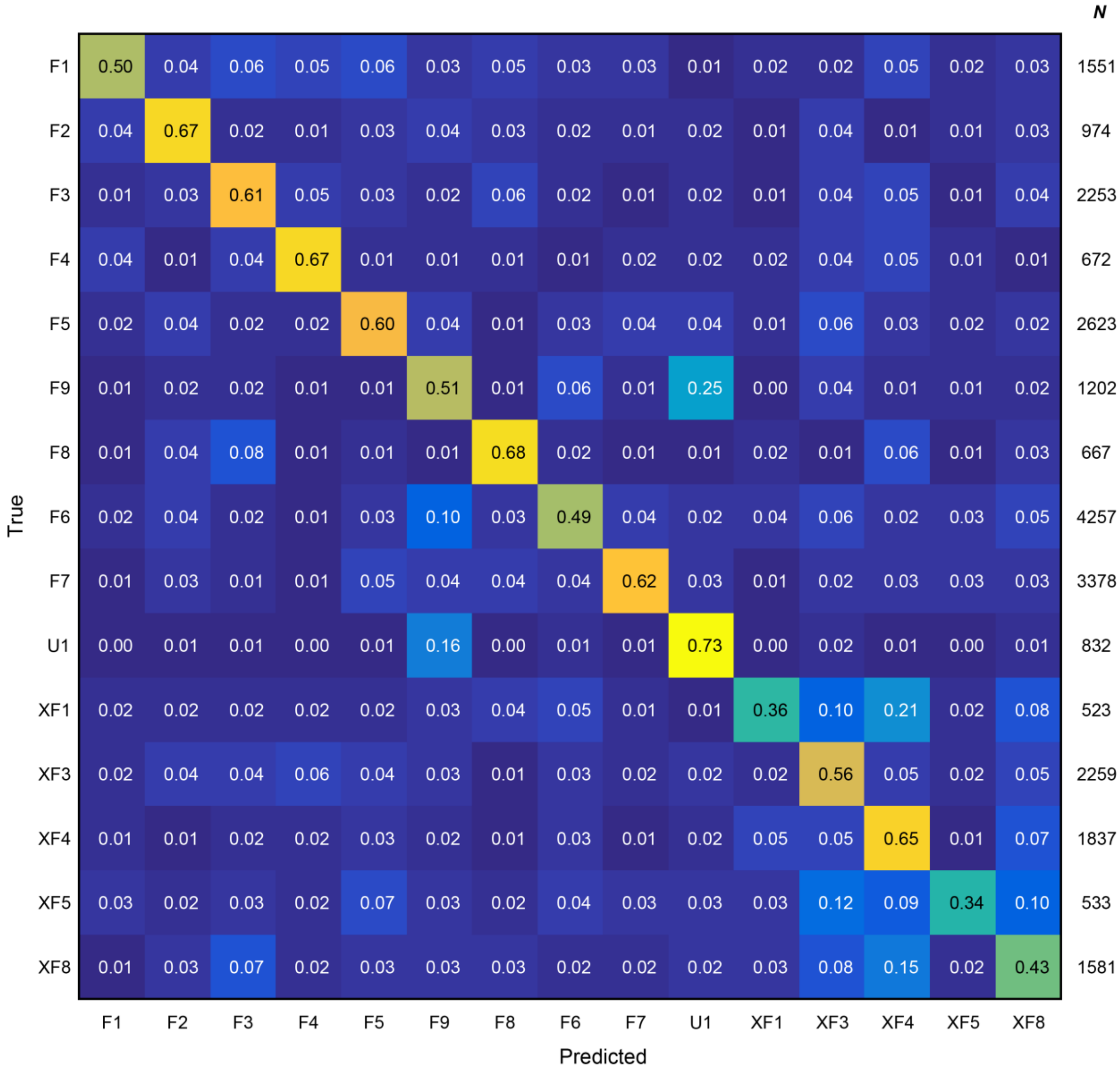

Figure S3. Emitter identification for an extended data. A confusion matrix (as described in the legend of Fig. S2) for the prediction of the emitting individual (BA=56%, chance=7%, p<0.01). This analysis was performed on all recordings from this study, including recordings conducted previously in the same setup, excluding pups and bats with less than 400 recorded vocalizations. This analysis indicates that emitter recognition is possible on larger numbers of individuals, and probably only depends on the amount of data available for the training procedure. Bat IDs as described in Table S1, and XFx – Female from previous recordings, XMx – Male from previous recordings.

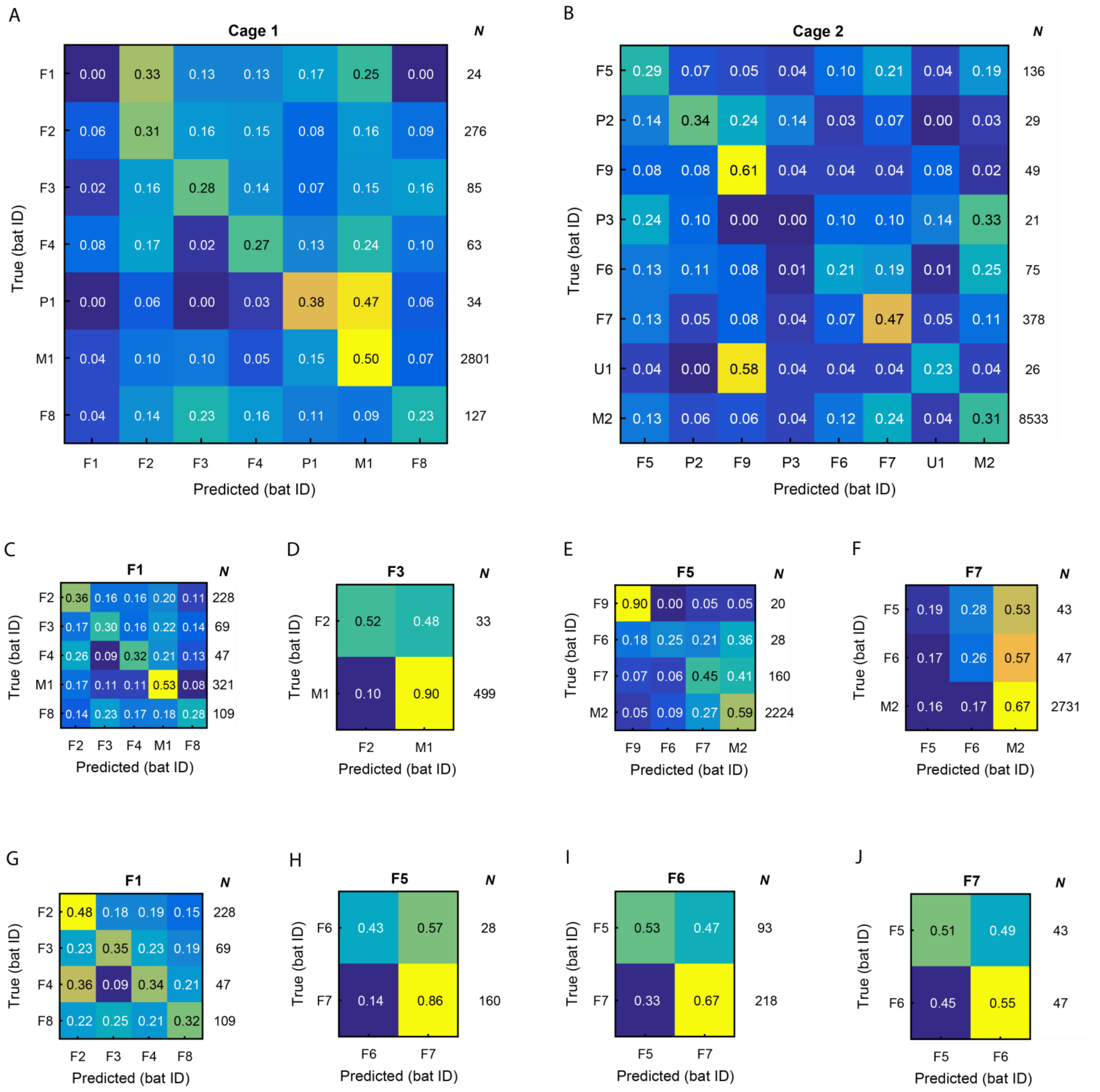

Figure S4. Prediction of addressees. Confusion matrices (as described in the legend of Fig. S2) for the prediction of the addressees of the vocalizations. (**A**) Addressees of the vocalizations in cage 1 (BA=28%, chance=14%, p<0.01). (**B**) Addressees of the vocalizations in cage 2 (BA=31%, chance=12.5%, p<0.01). (**C**) Addressees of vocalizations emitted by F1 (BA=36%, chance=20%, p<0.01). (**D**) Addressees of vocalizations emitted by F4 (BA=71%, chance=50%, p<0.01). (**E**) Addressees of vocalizations emitted by F5 (BA=55%, chance=25%, p<0.01). (**F**) Addressees of

vocalizations emitted by F7 (BA=37%, chance=33%, p=0.08). (**G**) Addressees of vocalizations emitted by F1 (excluding those directed to the male) (BA=37%, chance=25%, p<0.01). (**H**) Addressees of vocalizations emitted by F5 (excluding those directed to the male) (BA=64%, chance=50%, p=0.02). (**I**) Addressees of vocalizations emitted by F6 (excluding those directed to the male) (BA=60%, chance=50%, p<0.01). (**J**) Addressees of vocalizations emitted by F7 (excluding those directed to the male) (BA=53%, chance=50%, p=0.29).

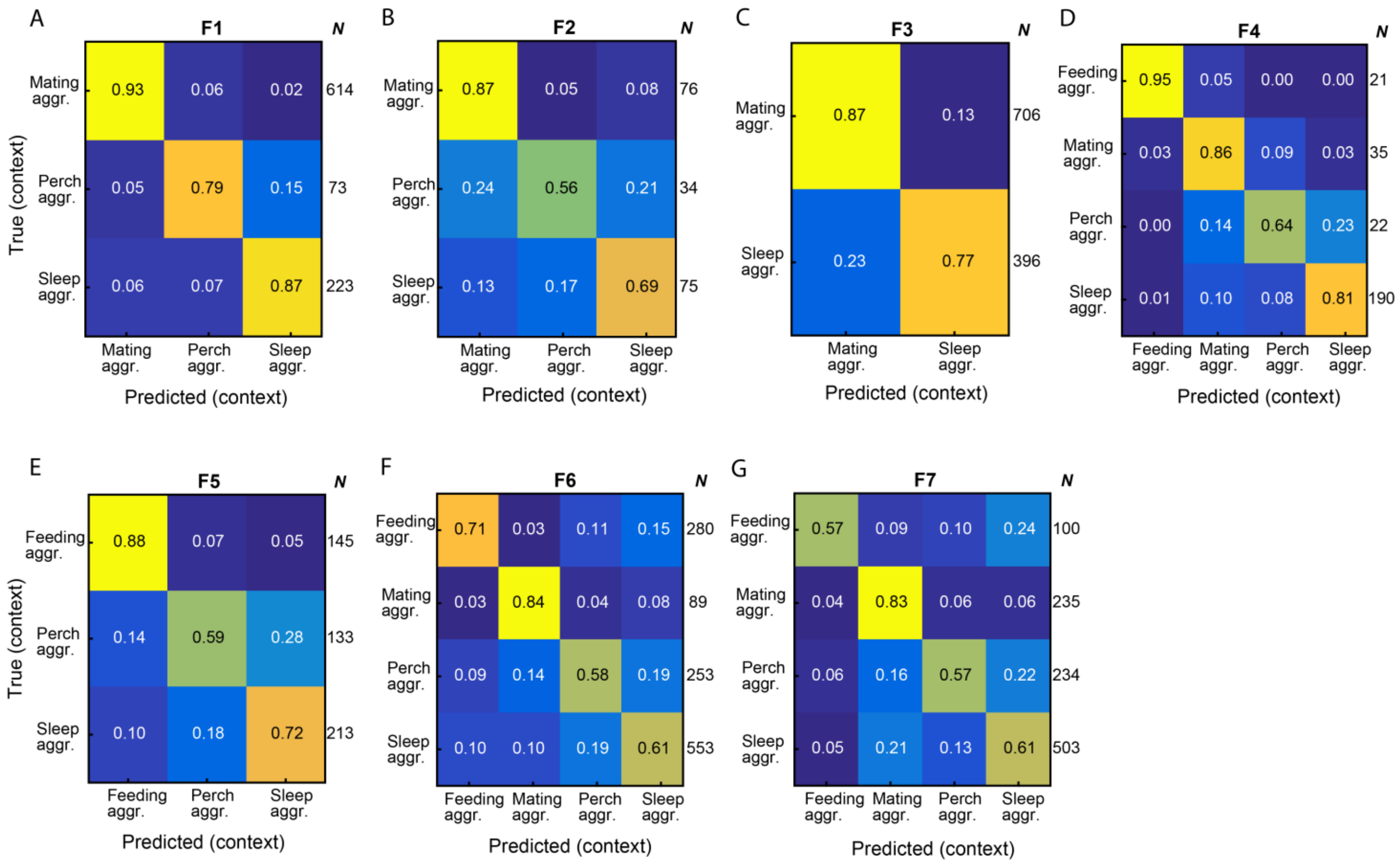

Figure S5. Context identification for specific emitters. Confusion matrices (as described in the legend of Fig. S2) for the prediction of the context for each individual separately. (**A**) Vocalizations produced by F1 (BA=86%, chance=33%, p<0.01). (**B**) Vocalizations produced by F2 (BA=71%, chance=33%, p<0.01). (**C**) Vocalizations produced by F3 (BA=82%, chance=50%, p<0.01). (**D**) Vocalizations produced by F4 (BA=81%, chance=25%, p<0.01). (**E**) Vocalizations produced by F5 (BA=73%, chance=33%, p<0.01). (**F**) Vocalizations produced by F6 (BA=69%, chance=25%, p<0.01). (**G**) Vocalizations produced by F7 (BA=65%, chance=25%, p<0.01).

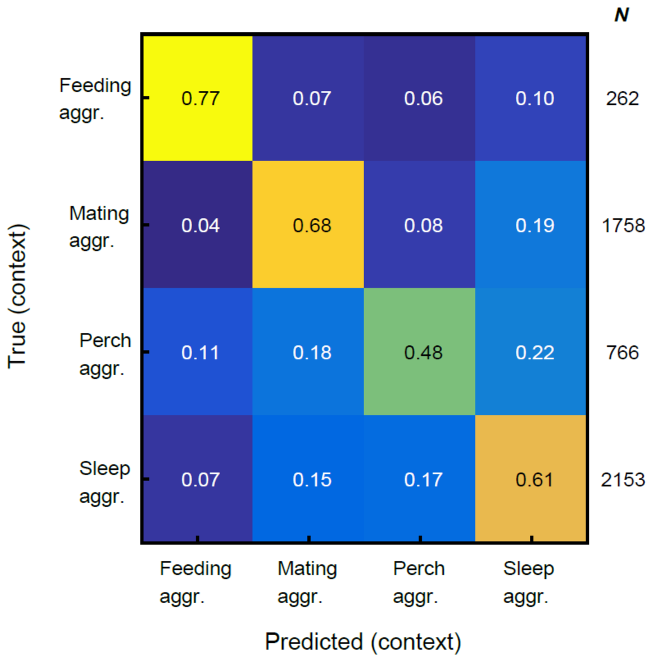

Figure S6. Context identification while controlling for possible food in the mouth of emitters of feeding aggressive vocalizations. To verify that classification of *feeding aggr.* context is not biased by the possible present of food in the mouth of the emitter, we sampled some of the videos and scrutinized them again to verify presence/absence of food. We then repeated the classification of the context using only interactions with no food in the mouth of the emitter (BA=64%, chance=25%, $p<0.01$).

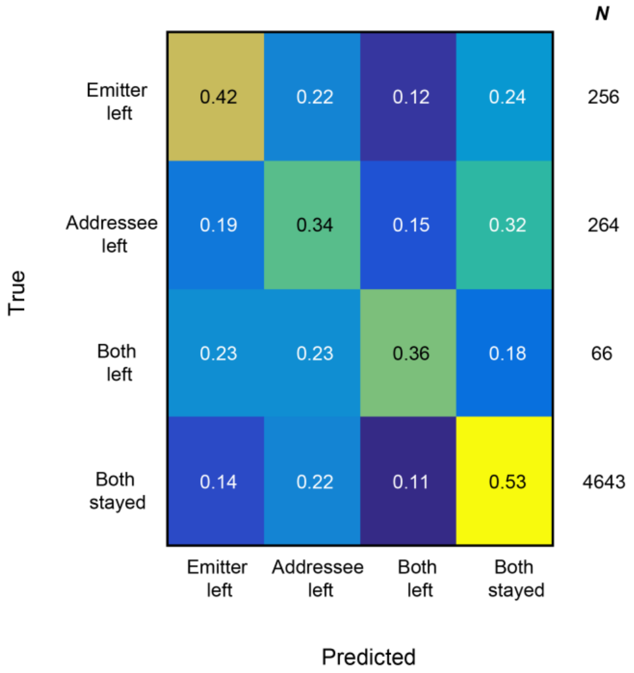

Figure S7. Predicting the detailed behavioral outcome of the interaction. A confusion matrix for the prediction of four possible outcomes: *Emitter left*, *Addressee left*, *both left*, and *both stayed* (BA=41%, chance=25%, p<0.01).

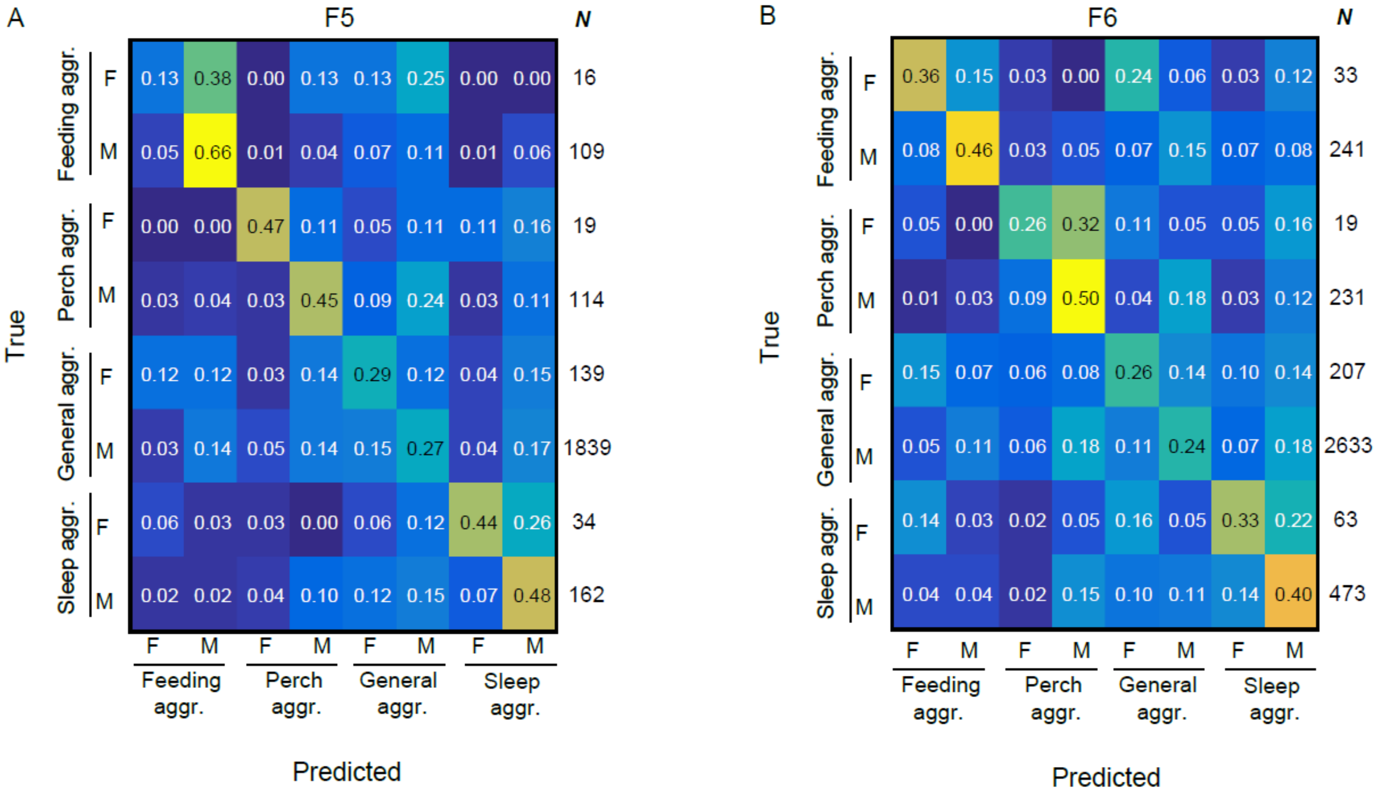

Figure S8. Classification of context and addressee's sex concurrently. A confusion matrix for the prediction of the behavioral context and the addressee's sex, for the two individual emitters for which all 8 combinations include at least 10 vocalizations. (**A**) Vocalizations emitted by bat F5 (BA=40%, chance=12.5%, p<0.01, permutation test). (**B**) Vocalizations emitted by bat F6 (BA=35%, chance=12.5%, p<0.01, permutation test). F – Female addressee, M – Male addressee.

Supplementary Movies

Video S1. Example of feeding aggression vocalizations. Two bats (hanging from the upper fruit skewer) are interacting during feeding. The emitting individual is marked with a red arrow.

Video S2. Example of mating aggression vocalizations. A female is protesting against a male mating attempt (a male is attempting to mount a female with a pup still attached to her). The emitting individual is marked with a red arrow.

Video S3. Example of perch aggression vocalizations. Two bats are interacting, with limited physical contact relative to other contexts, while perching in their artificial roost. In this aggressive display, the male is the aggressor and the female reacts and retreats. Another female is seen protecting her pup and sidesteps the squabble. The emitting individual is marked with a red arrow.

Video S4. Example of sleep aggression vocalization. A bat is vocalizing while in the daytime sleeping cluster. Notice the pups held under the wing of both females. The emitting individual is marked with a red arrow.

Supplementary Methods

Segmentation of raw recordings

Raw audio recordings were segmented into syllables and filtered to remove noises using an automated process as described in [36]. Vocalizations are bouts (sequences) of varying number of syllables. Sequences separated by a silence of more than 120*ms* were considered as separate vocalizations. The value of 120*ms* was obtained by assuming that the intra-bout silence intervals are normally distributed while the inter-bout silence intervals are exponentially distributed (a result of the assumption that the vocalization occurrences can be approximated by a Poisson process). Such 2-component distribution was fitted to the entire pool of interval durations, and the value, from which the likelihood of the exponential component was bigger than the Gaussian component, was chosen (Fig. S7).

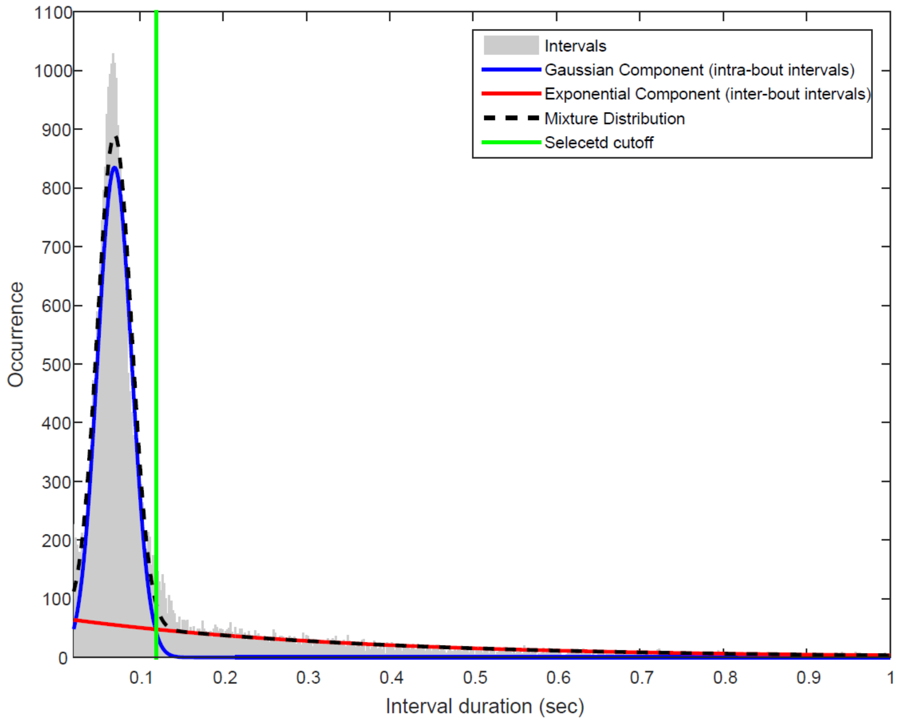

Fig. S7. Identification of inter-bout interval duration. A probability distribution was fitted to the empirical distribution of interval (between syllables) durations. The fitted distribution is a mixture of two components: Gaussian component for the intra-bout intervals, and exponential component for the inter-bout intervals. Intervals are considered as inter-bout where the density function of the exponential component is larger than that of the Gaussian component (from the green line onwards).

Classification

We used the GMM-UBM algorithm (following [39]).

We used Gaussian Mixtures Models (GMM) of 16 components in 64-dimensional space.

A Universal Background Model (UBM) was constructed by sampling 3900 syllables from data that was not used in the analysis. This sample was drawn from a set of vocalizations for which the pair of communicating bats was identified but it was not clear which was the emitter and which was the receiver. The syllables were randomly sampled in a balanced way, such that each individual was involved in the same number of interactions. A GMM was then fitted to this sample to create the UBM.

In the UBM-GMM approach, the training phase of the model fits a GMM to each class (e.g. each emitter, each context, etc.). However, instead of directly assessing the GMM parameters from the labeled data, the UBM is being used in an adaptive way [39].

Scoring test data. The score of a given test vocalization was computed for each class as the (log-) likelihood ratio between the class model (GMM) and the UBM. The class that received the highest score was the predicted class for this vocalization.
